# Prenatal exposure to inflammation increases anxiety-like behaviors in F1 and F2 generations: possible links to decreased FABP7 in hippocampus

**DOI:** 10.3389/fnbeh.2022.973069

**Published:** 2022-10-10

**Authors:** Jing Chen, Zhe-Zhe Zhang, Bao-Ling Luo, Qi-Gang Yang, Ming-Zhu Ni, Qi-Tao Wu, Yun Li, Xue-Wei Li, Gui-Hai Chen

**Affiliations:** ^1^Department of Neurology (Sleep Disorders), The Affiliated Chaohu Hospital of Anhui Medical University, Hefei, China; ^2^Department of Neurology or Department of Critical Care, The First Affiliated Hospital of Anhui Medical University, Hefei, China; ^3^Department of Neurology, The First Affiliated Hospital of Hengyang Medical School, University of South China, Hengyang, China

**Keywords:** aging, anxiety, hippocampus, FABP7, intergenerational transmission, mice, prenatal inflammation

## Abstract

Anxiety disorder has a high prevalence, and the risk of anxiety increases with age. Prenatal inflammation during key developmental timepoints can result in long-term changes in anxiety phenotype, even over a lifetime and across generations. However, whether maternal inflammation exposure during late gestation has intergenerational transmission effects on age-related anxiety-like behaviors and the possible underlying mechanisms are largely unknown. Fatty acid binding protein 7 (FABP7) is critical in hippocampal neurogenesis and is closely related to neuropsychiatric diseases, including anxiety disorder. The current study investigated the effects of maternal (F0 generation) lipopolysaccharide administration (50 μg/kg, i.p.) during late gestation on anxiety-like behaviors and FABP7 expression in F1 and F2 offspring, as well as the potential sex-specificity of intergenerational effects. Anxiety-like behaviors were evaluated using open field (OF), elevated plus maze, and black–white alley (BWA) tests at 3 and 13 months of age. The protein and messenger RNA levels of FABP7 in the hippocampus were measured using Western blot and real-time quantitative polymerase chain reaction (PCR), respectively. Overall, gestational LPS exposure in the F0 generation increased anxiety levels and decreased FABP7 expression levels in the F1 generation, which carried over to the F2 generation, and the intergenerational effects were mainly transferred *via* the maternal lineage. Moreover, hippocampal FABP7 expression was significantly correlated with performance in the battery of anxiety tests. The present study suggested that prenatal inflammation could increase age-related anxiety-like behaviors both in F1 and F2 offspring, and these effects possibly link to the FABP7 expression.

## Introduction

Anxiety disorders, some of the most common neuropsychiatric disorders, are highly prevalent in modern society. The interaction of environmental and genetic factors is thought to be involved in the etiology of anxiety disorders (Weissman et al., 2000; Hanamsagar and Bilbo, 2016). Accumulating evidence has indicated that prenatal developmental origins are relevant to neuropsychiatric disorders (Kinsella and Monk, 2009; Insel and Wang, 2010; O’Donnell and Meaney, 2017). This view has been encompassed in the “fetal origins hypothesis”, which states that a range of adverse exposures during the prenatal period can exert long-term negative effects on brain development and behaviors, with implications for mental and psychiatric health (Benarroch, 2013). Prenatal inflammation is a known etiological factor in most neuropsychiatric disorders. When an F0-generation pregnant mother is exposed to adverse conditions, both F1-generation embryos and the F2-generation germline are directly exposed (Bollati and Baccarelli, 2010). In animal models, adverse conditions in the uterus during germline programming may be transmitted through both paternal and maternal lines to induce disease phenotypes in offspring or even be inherited in multiple generations (Constantinof et al., 2016; Coley et al., 2019). Studies have revealed that the effects of chronic gestational inflammation on proinflammatory phenotype could be transmitted to F1 offspring and, to some extent, F2 offspring (Adams and Smith, 2019, 2020). Anxiety-like behaviors increase with age in rodents, for instance in Kunming, C57BL, and SAMP8 mice and rats (Chen et al., 2004; Schulz et al., 2007; Penteado et al., 2014). In our previous studies, increased anxiety with aging in SAMP8 mice was observed in elevated plus maze and black–white alley tasks, and a significant effect of age in CD-1 mice was observed in an open field and elevated plus maze tasks (Chen et al., 2007; Li et al., 2016). A growing body of literature shows that changes in anxiety-like behaviors reported in offspring are related to maternal systemic inflammation (Babri et al., 2014; Batinić et al., 2016; Li et al., 2021). Increasing evidence has indicated that gestational lipopolysaccharide (LPS) exposure can induce increased anxiety-like behaviors in adolescent and adult offspring (Enayati et al., 2012; Lin et al., 2012; Depino, 2015; Batinić et al., 2016). We previously found that maternal LPS exposure during late gestation augments age-related anxiety-like behaviors in F1 CD-1 mice (Li et al., 2016; Wang et al., 2020). However, it remains unknown whether grandmaternal LPS exposure during late pregnancy has effects on or causes intergenerational transmission of anxiety-like behaviors in F2 offspring and whether these effects derive from the paternal or maternal lineage (or both) in the F1 generation. Currently, very little data exists on the effects of F0-generation gestational LPS exposure on the anxiety levels of both F1 and F2 offspring in adolescence and midlife, respectively.

The hippocampus is a brain structure that plays an important role in memory and emotion (Drevets et al., 2008; Moorthi et al., 2015; Tsai et al., 2018; Rytova et al., 2019) and is particularly susceptible to inflammatory insult (Depino et al., 2005). Neuropsychiatric phenotypes associated with prenatal inflammation appear to be influenced by gestational timing, duration, and severity of infectious stressors, as well as the age and sex of offspring (Estes and McAllister, 2016; Brown and Meyer, 2018; Mac Giollabhui et al., 2019; Murray et al., 2019). In mice, hippocampal neurogenesis occurs mainly on gestational days 15, 16, and 17. The disruption of hippocampal neurogenesis associated with inflammation exposure might underlie behavioral manifestations of some diseases and contribute to behavioral disturbances that occur in aging (Chesnokova et al., 2016). Prenatal inflammation dampens hippocampal neurogenesis, which is associated with increased anxiety-like behaviors in adult offspring (Green and Nolan, 2014; Mouihate et al., 2019).

The content and dynamics of polyunsaturated fatty acids (PUFAs) in the brain can lead to changes in memory, learning, and emotional responses (Yamamoto and Owada, 2021). Fatty acid-binding proteins (FABPs), the cellular chaperones of PUFAs, are crucial in the uptake, transport, and storage of PUFAs (Furuhashi and Hotamisligil, 2008; Liu et al., 2010). The metabolism and activity of PUFAs play a vital role in the determination of mental state *via* FABPs (Matsumata et al., 2016). The brain-type FABP7 is a member of the intracellular lipid-binding protein family. Emerging research indicated that changes in FABP7 expression may be an underlying mechanism of neuropsychiatric disorders (Maekawa et al., 2011). For example, it has been reported that FABP7 is associated with anxiety in rodents and humans, that *Fabp7*-knockout mice exhibited anxiety-related traits in a series of behavioral tests, and that plasma FABP7 concentration in humans was significantly correlated with the severity of schizophrenia anxiety symptoms (Shimamoto et al., 2014; Koga et al., 2021). FABP7 expression follows the same temporal pattern as neurogenesis (Liu et al., 2010), and FABP7 levels begin to decline in early adulthood (Gerstner et al., 2008; Boneva et al., 2011; Foerster et al., 2020). In humans and rodents, hippocampal neurogenesis is critical for anxiety modulation (Revest et al., 2009; Fuss et al., 2010). Adult hippocampal neurogenesis is an important form of neuroplasticity that continuously declines with age in humans and rodents (Encinas et al., 2011; Foerster et al., 2020), and the process might be partially due to the loss of FABP7-expressing cells (Giachino et al., 2014).

We hypothesized that LPS exposure during late gestation may affect anxiety-like behaviors in both F1 and F2 offspring in adolescence and midlife, which may be related to altered expression of hippocampal FABP7 protein and messenger RNA (mRNA). In this study, we investigated: (a) whether gestational LPS exposure in the F0 generation could augment anxiety-like behaviors and hippocampal FABP7 expression levels alterations in F1 and F2 offspring in adolescence and midlife; (b) whether the differences in anxiety phenotypes and FABP7 expression of F2 offspring originate from F1 paternal or maternal lines; and (c) the association between anxiety-like behaviors and FABP7 expression.

## Materials and Methods

### Animals and experimental procedures

**Figure 1:**
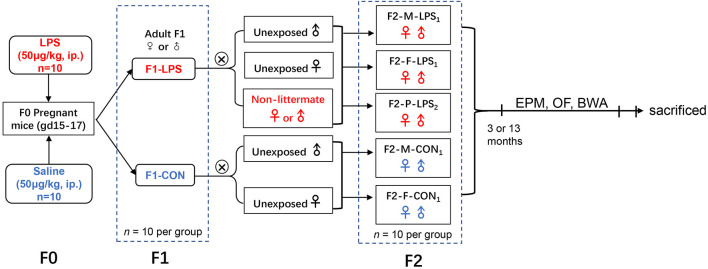
Timeline of experimental events. F2-M-LPS_1_, mice whose mothers were exposed to prenatal inflammation; F2-F-LPS_1_, mice whose fathers were exposed to prenatal inflammation; F2-P-LPS_2_, mice whose parents were exposed to prenatal inflammation; F2-M-CON_1_, mice whose mothers were exposed to saline *in utero*; F2-F-CON_1_, mice whose fathers were exposed to saline *in utero*; EPM, elevated plus maze; OF, open field; BWA, black–white alley; LPS, lipopolysaccharide; i.p., intraperitoneal; gd, gestational day.

CD-1 mice (6–8 weeks old) were purchased from the Beijing Vital River Laboratory Animal Technology Co., Ltd. (Beijing, P.R. China). Mice were housed in a controlled temperature (22°C–25°C) and humidity (50 ± 5%) environment with a 12-h light/dark cycle (lights on 7:00 a.m.). Food and water were freely provided. After 2 weeks of acclimation, female mice were paired with males at a 2:1 ratio. The emergence of a vaginal plug was designated as gestation day 0 (GD0). During GD15–17, pregnant mice (F0) received an intraperitoneal injection of LPS (50 μg/kg; Sigma-Aldrich, RRID:SCR_008988) or normal saline (control, CON). The first generation (F1), whose mothers were treated with LPS or saline, consisted of F1-LPS males (F1-LPS-M), F1-LPS females (F1-LPS-F), F1-CON males (F1-CON-M), and F1-CON females (F1-CON-F; *n* = 10 per group). F1 animals naturally mated with age-matched (2 months old) unexposed (been not descended from LPS or normal saline exposed dams) or non-littermate CD-1 mice to produce the second generation (F2). The F2 offspring consisted of the following groups: F2-**M**other-LPS_1_ (F2-M-LPS_1_), the offspring of F1-LPS females mated with unexposed males; F2-**F**ather-LPS_1_ (F2-F-LPS_1_), the offspring of F1-LPS males mated with unexposed females; F2-**P**arents-LPS_2_ (F2-P-LPS_2_), the offspring of F1-LPS females mated with non-littermate F1-LPS males; F2-**M**other-CON_1_ (F2-M-CON_1_), the offspring of F1-CON females mated with unexposed males; F2-**F**ather-CON_1_ (F2-F-CON_1_), the offspring of F1-CON males mated with unexposed females (*n* = 20 per group, ten males and ten females). Information on animal data is presented in Table 1. The F1 and F2 offspring were randomly selected to complete the elevated plus maze (EPM), open field (OF), and black–white alley (BWA) tests when they were 3 and 13 months old. The schematic representation of the experimental timeline is shown in Figure 1. All animal experiments were performed in compliance with the guidelines established by the National Institutes of Health Guide for the Care and Use of Laboratory Animals (National Research Council (US) Committee for the Update of the Guide for the Care and Use of Laboratory Animals, 2011). The protocol was approved by the Center for Laboratory Animal Sciences at Anhui Medical University (Hefei, P.R. China).

**Table 1:** Animal data in the study.

	**No. of animals**	**Mating partner**	**Total no. of offspring**	**Total male:female ratio**
F0 CON	10	-	98	54:44
F0 LPS	10	-	92	50:42
F1-CON-M	10	Unexposed female	106	49:57
F1-LPS-M	10	Unexposed female	95	55:40
F1-CON-F	10	Unexposed male	102	53:49
F1-LPS-F	10	Unexposed male	89	40:49
F1-LPS-F	10	F1-LPS-male	93	48:45

### Behavioral tests

#### Elevated plus maze

The EPM apparatus consisted of two opposite open arms (30×5 cm, without walls) and two opposite closed arms (30×5 cm) enclosed with walls (height 15 cm), forming the shape of a cross with a central arena (5×5 cm). The maze was placed 80 cm above the floor on a tripod. An overhead video camera was used to record behavior over a period of 6 min. For the observations, mice were individually placed on the central platform facing one of the open arms and were allowed to explore the maze for 6 min. The number of entries into the open arms and time spent on the open arms were recorded during a single trial. After each trial, the maze was cleaned with 70% ethanol before the next mouse was tested.

#### Open field

The OF apparatus was a black wooden box (81×81 cm, wall height 28 cm) with its floor divided into 16 equal-sized squares (each 20×20 cm). The squares were formed with white painted lines (width 3-mm) on the floor, and the central four squares were conceptually considered a central area; the other 12 squares along the walls were the peripheral area. Illumination was provided by a 40-W white light placed 2.80 m above the center of the field. For each trial, the animal was placed in a corner square, facing the wall, and was permitted to freely explore the environment for 5 min. Then, peripheral time (the time spent in the 12 peripheral squares), latency (the time before leaving the first square), and the number of squares crossed were recorded. The OF arena was cleaned with 70% ethanol between trials.

#### Black–White Alley (BWA)

For the Black–White Alley (BWA) apparatus, a narrow-galvanized iron box (120×30 cm, wall height 9 cm) without a top was used, of which one half was painted black and the other white. The two sections were connected, allowing the mice to move between the black and white sections. Each mouse was placed into the black half facing the wall in the apparatus. Mice were allowed to explore the two sections for 90 s, monitored by an automated video-tracking system. The following parameters were recorded: latency (time before entering the white section), time spent in the black section, and the number of transitions from the black to the white section. If the mouse never entered the white section, the latency was recorded as 90 s. The area was cleaned with 70% ethanol between trials.

### Tissue preparation

To avoid the possible influence of experimental manipulations on mRNA or protein expression, the mice were decapitated 2 weeks after the behavioral tests. Brains were promptly removed and bisected along the mid-sagittal suture on ice. The hippocampi were then rapidly isolated and frozen at −80°C for Western blotting and real-time quantitative polymerase chain reaction (PCR).

### Western blotting

Right hippocampal tissue was homogenized and centrifuged at 12,000 rpm for 10 min, and the supernatant was taken as the extracted protein. The protein concentration was measured using a bicinchoninic acid assay kit (Pierce Biotechnology, Waltham, MA, USA). Protein samples were separated in a 12.5% sodium dodecyl sulfate-polyacrylamide gel and then transferred onto polyvinylidene difluoride immunoblotting membranes by electrophoresis. To prevent non-specific binding of primary and/or secondary antibodies to the membrane, the membranes were blocked with 5% skim milk. The membranes were incubated with anti-rabbit FABP7 (1:1,000; Abcam, RRID:AB_880078) primary antibodies overnight at 4°C. After washing three times with PBS (Zsbio, ZLI-9062), the membranes were incubated with horseradish peroxidase-conjugated goat anti-rabbit IgG secondary antibodies (1:20,000; ZSGB Biotech, RRID:AB_2747412) for 2 h at room temperature and then washed three times. Immunoreactive bands at 15 kDa (FABP7) and 43 kDa (beta-actin, internal standard) denoted positive expression. Actin was used for normalization. Densitometric quantification of the band intensities was performed using ImageJ (Schneider et al., 2012). The ratio of the optical density of the anti-FABP7 antibody labeling to that of the anti-beta-actin antibody labeling in each sample was calculated as the relative FABP7 protein level. To control for equal loading, ratios of the optical density for the antibody of interest to the optical density of the antibody directed against beta-actin were calculated for each sample.

### Real-Time quantitative polymerase chain reaction

Left hippocampal tissue was homogenized and centrifuged at 12,000 rpm for 10 min at 4°C, isopropanol was added to the supernatant, and it was centrifuged at 12,000 rpm for 15 min at 4°C. The RNA pellet was washed two times and the supernatant was discarded. The extracted total RNA was stored at −80°C. The purity and content of the extracted RNA were assessed using a spectrophotometer. The RevertAid^TM^ First-Strand cDNA Synthesis Kit (Thermo Fisher Scientific, Waltham, MA, USA) was used to reverse transcribe RNA (1 μg) into complementary DNA. Real-time PCR was performed using Novostart SYBR qPCR SuperMix Plus, including 5 μl of 2× SYBR Green mix, 1 μl of primers (10 μM), 1 μl of complementary DNA template, and 2 μl of RNase-free water in a 10-μl reaction mixture. The quantitative real-time PCR reaction conditions were 95°C for 1 min (1 cycle); and 95°C for 20 s and 60°C for 1 min (40 cycles). The mRNA level was quantified using the 2^△△Ct^ method. Beta-actin served as the internal reference. The primer sequences are listed in Table 2.

**Table 2:** Sequences of the primers used for quantitative real-time polymerase chain reaction.

**Target genes**	**Amplicon size (bp)**	**Forward primer (5’→3’)**	**Reverse primer (5’→3’)**
β-actin	120	AGTGTGACGTTGACATCCGT	TGCTAGGAGCCAGAGCAGTA
FABP7	163	AGAAGTGGGATGGCAAAGAA	TAACTCTGGGACTCCAGGAA

### Statistical analysis

The data were presented as the mean ± standard error of the mean, and normality was examined with the Kolmogorov–Smirnov test. The effect of age was analyzed using two-way ANOVA with age and sex as independent variables in the control groups; the effect of LPS-affected was analyzed using two-way ANOVA with LPS-affected and sex as independent variables in the F1 and F2 dataset in each age group separately. A Fisher’s *post-hoc* analysis was performed to compare the results among the groups. *P* < 0.05 was used for statistical significance. The Pearson correlation test was conducted to analyze correlations between the relative levels of FABP7 in the hippocampus and behavioral test performance. Statistical analyses and figure production were performed using Graph Pad Prism (v8; Graph Pad Software Inc., RRID:SCR_002798).

## Results

### Increased anxiety-like behaviors in F1- and F2-generation mice

#### Elevated plus maze

##### Age effect

There were no significant differences for the 3- and 13-month CON groups on the number of open arm entries and the time spent on the open arm (*F*_(1,36)_ = 0.465, 0.180; *P* = 0.500, 0.674). No significant interaction of age × sex was observed on the EPM parameters (all *P* > 0.05).

##### LPS-affected effect

In the F1 generation, at 3 months of age, there was no significant effect of LPS-affected (*F*_(1,36)_ = 0.006, 0.074; *P* = 0.940, 0.787) or sex (*F*_(1,36)_ = 1.306, 0.003; *P* = 0.261, 0.958) on the number of open arm entries and the time on the open arm. There was no significant interaction between LPS-affected × sex (all *P* > 0.05). However, at the age of 13 months, there was a significant effect of LPS-affected (*F*_(1,36)_ = 8.414, *P* = 0.006) on the time on the open arm, with the female mice in the LPS group spending significantly less time on the open arm than those in the CON group (*P* = 0.013, Supplementary Table S1). Sex had no significant effect on the measured parameters (*F*_(1,36)_ = 0.080, 0.059; *P* = 0.778, 0.809). No significant interaction between age × sex was observed (all *P* > 0.05).

In the F2 generation, at the age of 3 months, there was no significant effect of LPS-affected (*F*_(4,90)_ = 0.300, 0.123; *P* = 0.877, 0.974), sex (*F*_(1,90)_ = 0.515, 1.338; *P* = 0.475, 0.251)or interaction of LPS-affected × sex (*F*_(4,90)_ = 0.050, 0.223; *P* = 0.995, 0.925) on the number of open arm entriesandthe time on the open arm. At the age of 13 months, the effects of LPS-affected (*F*_(4,90)_ = 0.510, 0.131; *P* = 0.728, 0.971) and sex (*F*_(1,90)_ = 0.162, 1.285; *P* = 0.688, 0.260), as well as the interaction of LPS-affected × sex (*F*_(4,90)_ = 0.308, 0.129; *P* = 0.872, 0.971) were not significant for mice.

#### Open field

##### Age effect

**Figure 2:**
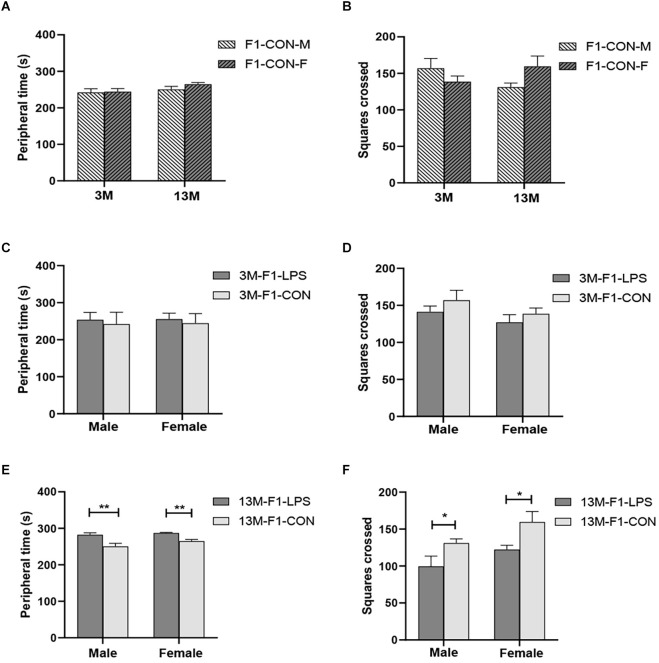
The results of the open field test in the F1 generation. **(A,B)** Peripheral time and the number of squares crossed at different ages in control groups. *n* = 10 male or female mice/group. Error bars = standard error of the mean (SEM); *P < 0.05, **P < 0.01 compared with the 3-month-old group. **(C,E)** Peripheral time and **(D,F)** number of squares crossed for 3- (3 M) and 13-month-old (13 M) CD-1 mice in the different treatment groups. *n* = 10 male or female mice/group. Error bars = SEM. *P < 0.05, **P < 0.01. CON-M, male mice exposed to saline *in utero*; CON-F, female mice exposed to saline *in utero*; F1-CON, F1 mice exposed to saline *in utero*; F1-LPS, F1 mice exposed to prenatal inflammation.

There was a significant age effect for the CON groups on the latency (*F*_(1,36)_ = 4.204, *P* = 0.048), but not on the peripheral time (*F*_(1,36)_ = 3.130, *P* = 0.085) and the number of squares crossed (*F*_(1,36)_ = 0.050, *P* = 0.824; Figures 2A,B). A significant interaction of age × sex on the number of squares crossed was observed (*F*_(1,36)_ = 4.612, *P* = 0.039). The effect of age is because the 13-month CON-F group had longer latency than the 3-month CON-F group (*P* = 0.013, Supplementary Table S1).

##### LPS-affected effect

In the F1 generation, at 3 months of age, there was no significant effect of LPS-affected or sex on the peripheral time (*F*_(1,36)_ = 2.201, 0.071;*P* = 0.147, 0.791), the number of squares crossed (*F*_(1,36)_ = 1.792, 2.540; *P* = 0.189, 0.120) and the latency (*F*_(1,36)_ = 0.979, 0.371; *P* = 0.330, 0.547; Figures 2C,D and Supplementary Table S1). The interaction of LPS-affected × sex had no significant effect on the parameters (all *P* > 0.05). However, at 13 months of age, a significant effect of LPS-affected on the periphery time (*F*_(1,36)_ = 23.310, *P* < 0.001) and the number of squares crossed (*F*_(1,36)_ = 10.300, *P* = 0.003) was found, and the effect of sex was significant on the number of squares crossed (*F*_(1,36)_ = 5.744, *P* = 0.022). No significant interaction of LPS-affected × sex was found (all *P* > 0.05). Further, the F1-LPS offspring spend a longer time in the periphery and crossed fewer squares than the same-sex controls (all *P* < 0.05, Figures 2E,F).

**Figure 3:**
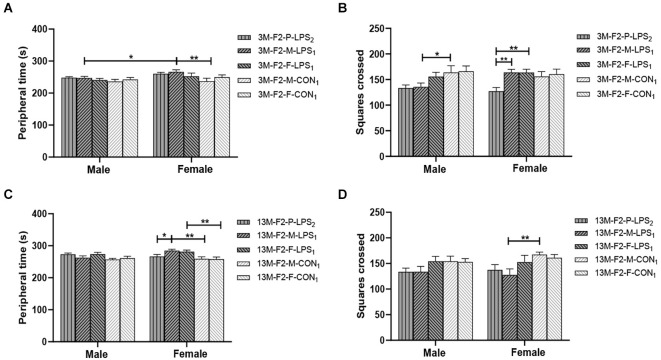
Results of the open field test in the F2 generation. **(A,C)** Peripheral time, **(B,D)** number of squares crossed in the 3-month-old (3 M) and 13-month-old (13 M) CD-1 mice in different groups. *n* = 10 male or female mice/group. Error bars = standard error of the mean (SEM). *P < 0.05, **P < 0.01; F2-M-LPS_1_, mice whose mothers were exposed to prenatal inflammation; F2-P-LPS_2_, mice whose parents were exposed to prenatal inflammation; F2-F-LPS_1_, mice whose fathers were exposed to prenatal inflammation; F2-M-CON_1_, mice whose mothers were exposed to saline* in utero*; F2-F-CON_1_, mice whose fathers were exposed to saline *in utero*.

In the F2 generation, at the age of 3 months, the effect of LPS-affected was significant on the peripheral time (*F*_(4,90)_ = 2.822, *P* = 0.030), and the number of squares crossed (*F*_(4,90)_ = 4.713, *P* = 0.002), but not on the latency (*F*_(4,90)_ = 2.102, *P* = 0.087). We found a significant main effect of sex on the peripheral time (*F*_(1,90)_ = 5.913, *P* = 0.017), with the M-LPS_1_ female mice had longer peripheral time than their counterpart male mice (*P* = 0.043; Figure 3A). There was no significant interaction between LPS-affected × sex (all *P* > 0.05). The main effect of LPS-affected indicated that the M-LPS_1_ (LPS-affected mothers) female mice had longer peripheral time relative to the M-CON_1_ (unaffected mothers) female mice (*P* = 0.003; Figure 3A), the M-LPS_1_ male mice had fewer squares crossed relative to the M-CON_1_ male mice (*P* = 0.025; Figure 3B). Besides, the P-LPS_2_ female mice crossed a lower number of squares than the M-LPS_1_ and F-LPS_1_ female mice (all *P* < 0.01; Figure 3B). At the age of 13 months, there was a significant effect of LPS-affected on the peripheral time (*F*_(4,90)_ = 4.942, *P* = 0.001), the latency (*F*_(4,90)_ = 2.921, *P* = 0.025), and the number of squares crossed (*F*_(4,90)_ = 4.132, *P* = 0.004). The effect of sex on the peripheral time (*F*_(1,90)_ = 1.645, *P* = 0.203), the latency (*F*_(1,90)_ = 0.020, *P* = 0.888), and the number of squares crossed (*F*_(1,90)_ = 0.337, *P* = 0.563) was not significant. There was no significant interaction between LPS-affected × sex (all *P* > 0.05). The main effect of LPS-affected is because longer peripheral time was observed in the female mice from the M-LPS_1_ and F-LPS_1_ (LPS-affected fathers) groups compared to the female mice from the corresponding CON (unaffected mothers or fathers) groups (*P* = 0.001, 0.004; Figure 3C); longer peripheral time was observed in the M-LPS_1_ female mice compared to the P-LPS_2_ (LPS-affected parents) female mice (*P* = 0.019; Figure 3C). The M-LPS_1_ mice had longer latency relative to the same-sex M-CON_1_ mice (*P* = 0.042, 0.048; Supplementary Table S1), the M-LPS_1_ female mice had fewer squares crossed relative to the M-CON_1_ female mice (*P* = 0.004; Figure 3D).

#### Black–White Alley

##### Age effect

**Figure 4:**
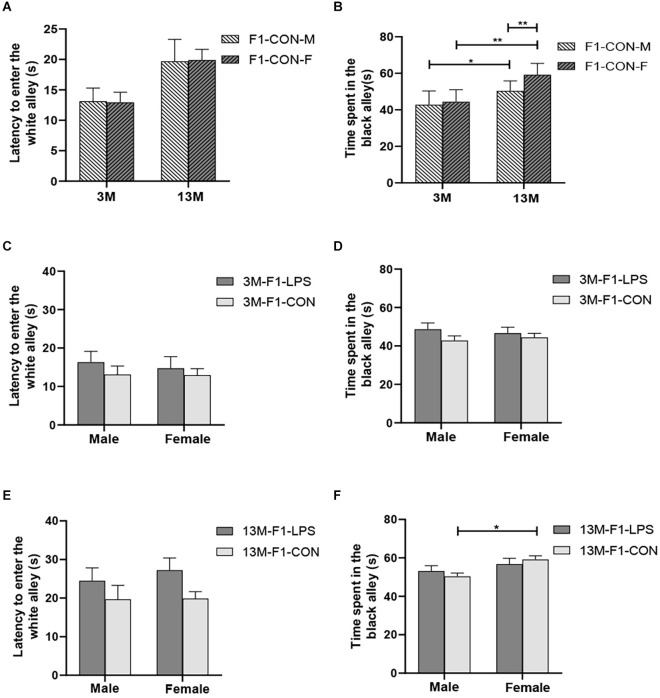
The results of the black–white alley test in the F1 generation. **(A,B)** Latency before entering the white alley, time spent in the black alley at different ages in the control groups. *n* = 10 male or female mice/group. Error bars = standard error of the mean (SEM); *P < 0.05, **P < 0.01 compared with the 3-month-old (3 M) group. **(C,E)** Latency before entering the white alley and **(D,F)** time spent in the black alley for 3- (3 M) and 13-month-old (13 M) CD-1 mice in the different treatment groups; *n* = 10 male or female mice/group. Error bars = SEM. *P < 0.05. CON-M, male mice exposed to saline *in utero*; CON-F, female mice exposed to saline *in utero*; F1-CON, F1 mice exposed to saline *in utero*; F1-LPS, F1 mice exposed to prenatal inflammation.

There was a significant age effect for the 3- and 13-month CON groups on the latency before entering the white alley and the time spent in the black alley (*F*_(1,36)_ = 7.71, 629.130; *P* = 0.009, < 0.001; Figures 4A,B), but not on the number from black to the white alley (*F*_(1,36)_ = 2.684; *P* = 0.110; Supplementary Table S1). There was no significant interaction between LPS-affected × sex (all *P* > 0.05). Further, longer time spent in the black alley was observed in the 13-month CON mice compared to the same-sex 3-month CON mice (*P* = 0.014, < 0.001; Figure 4B).

##### LPS-affected effect

In the F1 generation, at 3 months of age, there was no significant effect of LPS-affected or sex on the latency before entering the white alley (*F*_(1,36)_ = 0.988, 0.123; *P* = 0.327, 0.727), the time spent in the black alley (*F*_(1,36)_ = 2.105, 0.008; *P* = 0.156, 0.927), and the number from black to white alley (*F*_(1,36)_ = 0.470, 3.066; *P* = 0.498, 0.089; Figures 4C,D and Supplementary Table S1). No significant interaction of LPS-affected × sex was observed (all *P* > 0.05). At 13 months of age, a significant effect of sex on the time spent in the black alley (*F*_(1,36)_ = 6.408, *P* = 0.016) and the number from black to white alley (*F*_(1,36)_ = 6.069, *P* = 0.019) was found, but the effect of LPS-affected on the latency before entering the white alley (*F*_(1,36)_ = 3.989; *P* = 0.053), the time spent in the black alley (*F*_(1,36)_ = 0.007; *P* = 0.933), and the number from black to white alley (*F*_(1,36)_ = 0.075; *P* = 0.786) was not significant (Figure 4E). There was no significant interaction between LPS-affected × sex (all *P* > 0.05). The effect of sex showed that more time spent in the black alley in CON female mice relative to CON male mice (*P* = 0.015; Figure 4F) and fewer transitions in LPS female mice relative to LPS male mice (*P* = 0.014; Supplementary Table S1).

**Figure 5:**
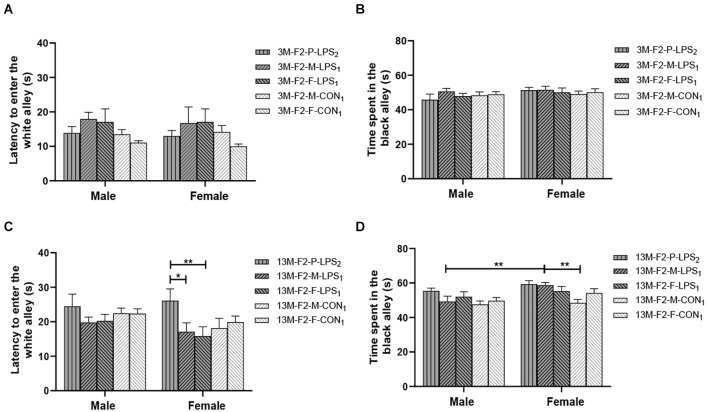
Results of the black–white alley test in the F2 generation. **(A,C)** Latency before entering the white alley and **(B,D)** time spent in the black alley in the 3- (3 M) and 13-month-old (13 M) CD-1 mice in different groups. *n* = 10 male or female mice/group. Error bars = SEM. *P < 0.05, **P < 0.01; F2-M-LPS_1_, mice whose mothers were exposed to prenatal inflammation; F2-P-LPS_2_, mice whose parents were exposed to prenatal inflammation; F2-F-LPS_1_, mice whose fathers were exposed to prenatal inflammation; F2-M-CON_1_, mice whose mothers were exposed to saline *in utero*; F2-F-CON_1_, mice whose fathers were exposed to saline *in utero*.

In the F2 generation, in the 3-month-old mice, there was no significant effect of LPS-affected on the latency before entering the white alley (*F*_(4,90)_ = 2.433; *P* = 0.053), the time spent in the black alley (*F*_(4,90)_ = 0.485; *P* = 0.746), and the number from black to white alley (*F*_(4,90)_ = 1.848; *P* = 0.127). The effect of sex on the latency before entering the white alley (*F*_(1,90)_ = 0.087, *P* = 0.769), the time spent in the black alley (*F*_(1,90)_ = 2.688, *P* = 0.105), and the number from black to white alley (*F*_(1,90)_ = 0.390, *P* = 0.534) was not significant (Figures 5A,B and Supplementary Table S1). The interaction of LPS-affected × sex had no significant effect on these parameters (all *P* > 0.05). In the 13-month-old mice, LPS-affected had a significant effect on the latency before entering the white alley (*F*_(4,90)_ = 2.805, *P* = 0.030) and the time spent in the black alley (*F*_(4,90)_ = 4.291, *P* = 0.003), but not on the number from black to white alley (*F*_(4,90)_ = 0.569, *P* = 0.686). There was no significant interaction between LPS-affected × sex (all *P* > 0.05). Further, the effect of LPS-affected indicated that the female mice in the P-LPS_2_ group had longer latencies than those in the M-LPS_1_ and F-LPS_1_ groups (*P* = 0.011, 0.004; Figure 5C), and the female M-LPS_1_ mice spent more time in the black alley than the female M-CON_1_ mice (*P* = 0.002; Figure 5D). Additionally, there was a significant effect of sex on the time spent in the black alley (*F*_(1,90)_ = 9.004, *P* = 0.004), with more time spent in the black alley in the female M-LPS_1_ mice relative to the male M-LPS_1_ mice (*P* = 0.005; Figure 5D).

#### Summary

The results from the F1 and F2 generations in the battery of anxiety tasks are summarized in Supplementary Table S1.

### Decreased FABP7 protein and mRNA expression in the F1 and F2 generations

#### Age effect

**Figure 6:**
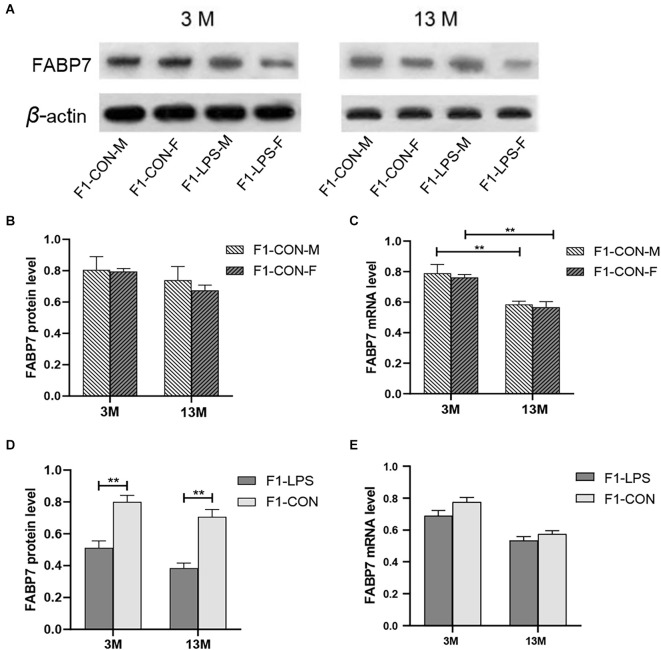
FABP7 protein and mRNA expression levels in F1 generation mice. **(A)** Representative FABP7 immunoreactive bands in the hippocampi of F1 mice in the different groups at different ages. **(B,C)** FABP7 protein and mRNA levels in the hippocampi of mice at different ages in the control groups; *n* = 6 male or female mice/group. Error bars = standard error of the mean (SEM). **P < 0.01 compared with the 3 M group. **(D)** FABP7 protein and **(E)** FABP7 mRNA levels in the hippocampi for 3- (3 M) and 13-month-old (13 M) CD-1 mice in different groups; *n* = 12 per group. Error bars = SEM. **P < 0.01. CON-M, male mice exposed to saline *in utero*; CON-F, female mice exposed to saline *in utero*; F1-CON, F1 mice exposed to saline *in utero*; F1-LPS, F1 mice exposed to prenatal inflammation.

Representative FABP7 immunoreactive bands in the hippocampi of F1 mice at 3- and 13-month-old are shown in Figure 6A. We found a significant main effect of age for the CON group on hippocampal FABP7 mRNA levels (*F*_(1,20)_ = 29.36; *P* < 0.01), but not on FABP7 protein levels (*F*_(1,20)_ = 2.151; *P* = 0.158; Figure 6B). The interaction of age × sex had no significant effect on FABP7 protein and mRNA expression (all *P* > 0.05). The age effect indicated that lower mRNA levels in the older CON mice in both sexes were found relative to the corresponding 3-month-old mice (all *P* < 0.001; Figure 6C).

#### LPS-affected effect

In the F1 generation, in the 3-month-old mice, there was a significant effect of LPS-affected on the FABP7 protein levels (*F*_(1,20)_ = 22.45; *P* = 0.001), with lower protein levels were found in the LPS group compared to the corresponding CON group (*P* < 0.01; Figure 6D), but not on the FABP7 mRNA levels (*F*_(1,20)_ = 3.637; *P* = 0.071; Figure 6E). The sex had no significant effect on the protein and mRNA levels (*F*_(1,20)_ = 0.569, 0.694; *P* = 0.459, 0.415). There was no significant interaction between LPS-affected × sex (all *P* > 0.05). Similarly, in the 13-month-old mice, the effect of LPS-affected was significant on the protein levels (*F*_(1,20)_ = 33.210; *P* < 0.001) but not on the mRNA levels (*F*_(1,20)_ = 2.030; *P* = 0.170; Figure 6E). No significant interaction of LPS-affected × sex was observed (all *P* > 0.05). Further, lower protein levels in the LPS mice were found relative to the corresponding CON mice (*P* < 0.001; Figure 6D).

**Figure 7:**
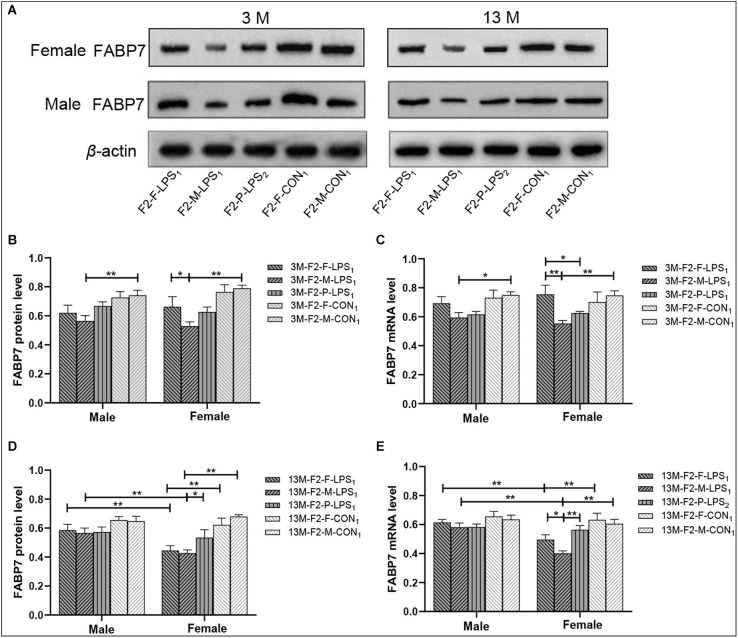
FABP7 protein and mRNA expression levels in F2 generation mice. **(A)** Representative FABP7 immunoreactive bands in the hippocampi of F2 mice in the different groups at different ages. **(B,D)** Hippocampal FABP7 protein levels in 3-month-old (3 M) and 13-month-old (13 M) mice in different groups. **(C,E)** Hippocampal FABP7 mRNA levels in the 3-month-old (3 M) and 13-month-old (13 M) mice in different groups; *n* = 6 male or female mice/group. Error bars = standard error of the mean (SEM). *P < 0.05, **P < 0.01. F2-M-LPS_1_, mice whose mothers were exposed to prenatal inflammation; F2-P-LPS_2_, mice whose parents were exposed to prenatal inflammation; F2-F-LPS_1_, mice whose fathers were exposed to prenatal inflammation; F2-M-CON_1_, mice whose mothers were exposed to saline *in utero*; F2-F-CON_1_, mice whose fathers were exposed to saline *in utero*.

Representative FABP7 immunoreactive bands in the hippocampi of F2 mice at 3- and 13-month-old are shown in Figure 7A. In the F2 generation, in 3-month-old mice, a significant effect of LPS-affected was observed in both FABP7 protein and mRNA levels (*F*_(4,50)_ = 8.832, 6.529; *Ps* < 0.001), the mice from the M-LPS_1_ group showed lower FABP7 protein and mRNA levels than those from the M-CON_1_ group (all *P* < 0.01; Figures 7B,C), the M-LPS_1_ female mice hadlower protein and mRNA levels relative to the F-LPS_1_ female mice (all *P* < 0.05; Figures 7B,C) and the P-LPS_2_ female mice had lower mRNA levels than the F-LPS_1_ female mice (*P* = 0.033; Figure 7C). The effect of sex on the FABP7 protein and mRNA levels was not significant (*F*_(1,50)_ = 0.156, < 0.001; *P* = 0.695, 0.989). The interaction of LPS-affected × sex had no significant effect on the protein and mRNA levels (all *P* > 0.05). In the 13-month-old mice, we found significant effects of LPS-affected (*F*_(4,50)_ = 8.693, 7.896; *Ps* < 0.001) and sex (*F*_(1,50)_ = 8.057, 15.110; *Ps* < 0.01) on both FABP7 protein and mRNA levels. The interaction of LPS-affected × sex had a significant effect on mRNA levels (*F*_(4,50)_ = 2.891; *P* = 0.031). Further, lower protein and mRNA levels were seen in the female mice from the M-LPS_1_ and F-LPS_1_ groups compared to the corresponding CON groups (all *P* < 0.01; Figures 7D,E). The M-LPS_1_ female mice had significantly lower protein levels than the P-LPS_2_ female mice (*P* = 0.037; Figure 7D) and had lower mRNA levels relative to the F-LPS_1_ and P-LPS_2_ female mice (all *P* < 0.05; Figure 7E). The effect of sex occurred in the M-LPS_1_ and F-LPS_1_ groups, with lower FABP7 protein and mRNA levels in female mice relative to male mice (all *P* < 0.01; Figures 7D,E).

### Correlations between anxiety-like behaviors and FABP7 protein and mRNA expression in F1 and F2 generations

The results of correlations between performance in the anxiety-related tasks and hippocampal FABP7 expression in F1 and F2 offspring are shown in Tables s 3, 4, respectively.

**Table 3:** Correlations between performance in anxiety-related tasks and hippocampal FABP7 protein and mRNA levels in F1 offspring.

**Tasks**	**Measures**	**Ages**	**Groups**	**FABP7**	
				**Protein [*r*(*p*)]**	**mRNA [*r*(*p*)]**
Open Field	Peripheral time	3 months	LPS-M	0.348 (0.499)	−0.398 (0.434)
			LPS-F	0.010 (0.985)	−0.283 (0.586)
			CON-M	−0.224 (0.669)	−0.083 (0.876)
			CON-F	0.052 (0.922)	−0.563 (0.245)

		13 months	LPS-M	−0.774 (0.071)	−0.873 (0.023)*
			LPS-F	−0.916 (0.010)*	−0.920 (0.009)**
			CON-M	0.512 (0.300)	0.636 (0.175)
			CON-F	0.051 (0.923)	−0.112 (0.833)

	Squares crossed	3 months	LPS-M	−0.572 (0.235)	−0.637 (0.173)
			LPS-F	−0.208 (0.692)	−0.008 (0.988)
			CON-M	0.022 (0.967)	0.076 (0.886)
			CON-F	0.165 (0.755)	−0.191 (0.716)

		13 months	LPS-M	0.826 (0.043)*	0.859 (0.029)*
			LPS-F	0.859 (0.028)*	0.874 (0.023)*
			CON-M	0.862 (0.027)*	0.960 (0.002)**
			CON-F	−0.281 (0.589)	−0.181 (0.731)

Black–white alley	Latency to enter the white alley	3 months	LPS-M	−0.687 (0.132)	−0.022 (0.967)
			LPS-F	0.570 (0.238)	−0.188 (0.722)
			CON-M	0.357 (0.487)	−0.186 (0.724)
			CON-F	0.017 (0.975)	0.494 (0.319)

		13 months	LPS-M	−0.914 (0.011)*	−0.888 (0.018)*
			LPS-F	−0.291 (0.576)	−0.068 (0.898)
			CON-M	−0.703 (0.119)	−0.976 (0.001)**
			CON-F	−0.819 (0.046)*	−0.792 (0.061)

	Time spent on the black alley	3 months	LPS-M	0.711 (0.113)	0.285 (0.585)
			LPS-F	−0.359 (0.484)	0.443 (0.379)
			CON-M	0.671 (0.144)	−0.806 (0.053)
			CON-F	0.066 (0.902)	0.266 (0.611)

		13 months	LPS-M	−0.827 (0.042)*	−0.941 (0.005)**
			LPS-F	−0.815 (0.048)*	−0.725 (0.103)
			CON-M	−0.896 (0.016)*	−0.890 (0.018)*
			CON-F	−0.922 (0.009)**	−0.877 (0.022)*

*P < 0.05; **P < 0.01.

**Table 4:** The correlations between performance in anxiety-related tasks and hippocampal FABP7 protein and mRNA levels in F2 offspring.

**Tasks**	**Measures**	**Ages**	**Groups**	**FABP7**			
				**Female**		**Male**	
				**protein [*r(p)*]**	**mRNA [*r(p)*]**	**protein [*r(p)*]**	**mRNA [*r(p)*]**
Open Field	Peripheral time	3 months	P-LPS_2_	0.644 (0.168)	0.584 (0.223)	−0.896 (0.016)*	−0.467 (0.351)
			M-LPS_1_	−0.888 (0.018)*	−0.851 (0.032)*	−0.902 (0.014)*	−0.875 (0.022)*
			F-LPS_1_	−0.749 (0.086)	−0.834 (0.039)*	−0.237 (0.651)	−0.371 (0.470)
			M-CON_1_	0.198 (0.706)	−0.086 (0.872)	0.354 (0.491)	0.575 (0.233)
			F-CON_1_	−0.085 (0.873)	−0.159 (0.764)	−0.216 (0.681)	0.178 (0.736)

		13 months	P-LPS_2_	−0.096 (0.857)	0.197 (0.708)	−0.508 (0.304)	−0.279 (0.592)
			M-LPS_1_	−0.945 (0.004)**	−0.805 (0.053)	0.247 (0.637)	0.489 (0.325)
			F-LPS_1_	−0.519 (0.292)	−0.427 (0.398)	0.310 (0.550)	0.115 (0.829)
			M-CON_1_	−0.939 (0.005)**	−0.843 (0.035)*	0.191 (0.717)	0.394 (0.440)
			F-CON_1_	0.559 (0.249)	0.429 (0.396)	0.810 (0.051)	0.133 (0.802)

	Squares crossed	3 months	P-LPS_2_	0.102 (0.848)	−0.116 (0.827)	−0.538 (0.271)	0.143 (0.788)
			M-LPS_1_	0.900 (0.015)*	0.962 (0.002)**	0.831 (0.040)*	0.928 (0.008)**
			F-LPS_1_	0.835 (0.039)*	0.675 (0.141)	0.738 (0.094)	0.894 (0.016)*
			M-CON_1_	0.945 (0.004)**	0.836 (0.038)*	−0.104 (0.844)	−0.364 (0.477)
			F-CON_1_	0.065 (0.902)	0.268 (0.607)	0.102 (0.847)	0.477 (0.338)

		13 months	P-LPS_2_	0.893 (0.017)*	0.855 (0.030)*	0.886 (0.019)*	0.925 (0.008)**
			M-LPS_1_	0.956 (0.003)**	0.826 (0.043)*	0.992 (0.000)**	0.931 (0.007)**
			F-LPS_1_	0.553 (0.255)	0.451 (0.370)	0.987 (0.000)**	0.976 (0.001)**
			M-CON_1_	−0.458 (0.361)	−0.213 (0.685)	0.767 (0.075)	0.453 (0.367)
			F-CON_1_	0.076 (0.887)	0.231 (0.660)	0.290 (0.577)	−0.316 (0.541)

Black–white alley	Latency to enter the white alley	3 months	P-LPS_2_	−0.088 (0.868)	−0.107 (0.840)	0.048 (0.928)	−0.412 (0.417)
			M-LPS_1_	−0.590 (0.217)	−0.797 (0.058)	−0.842 (0.035)*	−0.763 (0.078)
			F-LPS_1_	0.180 (0.733)	−0.835 (0.039)*	0.007 (0.990)	0.702 (0.120)
			M-CON_1_	0.084 (0.874)	−0.454 (0.365)	0.346 (0.501)	0.082 (0.878)
			F-CON_1_	0.065 (0.902)	0.628 (0.181)	−0.003 (0.996)	−0.540 (0.269)

		13 months	P-LPS_2_	0.049 (0.927)	−0.182 (0.730)	−0.978 (0.001)**	−0.782 (0.066)
			M-LPS_1_	−0.789 (0.062)	−0.913 (0.011)*	−0.957 (0.003)**	−0.855 (0.030)*
			F-LPS_1_	0.021 (0.968)	−0.213 (0.685)	0.182 (0.730)	−0.060 (0.911)
			M-CON_1_	−0.798 (0.057)	−0.659 (0.154)	−0.297 (0.568)	0.316 (0.541)
			F-CON_1_	−0.461 (0.358)	−0.246 (0.638)	−0.683 (0.135)	−0.319 (0.537)

	Time spent in the black alley	3 months	P-LPS_2_	−0.147 (0.781)	−0.352(0.493)	−0.361 (0.481)	−0.343 (0.506)
			M-LPS_1_	−0.358 (0.486)	−0.661 (0.153)	−0.884 (0.019)*	−0.864 (0.026)*
			F-LPS_1_	−0.466 (0.352)	0.131 (0.804)	−0.023 (0.966)	−0.356 (0.488)
			M-CON_1_	−0.636 (0.174)	0.097 (0.855)	−0.720 (0.107)	0.205 (0.697)
			F-CON_1_	−0.927 (0.008)**	0.044 (0.935)	−0.750 (0.086)	0.118 (0.824)

		13 months	P-LPS_2_	−0.051 (0.923)	0.114 (0.830)	−0.395 (0.438)	−0.333 (0.519)
			M-LPS_1_	−0.869 (0.024)*	−0.906 (0.013)*	−0.925 (0.008)**	−0.876 (0.022)*
			F-LPS_1_	−0.933 (0.007)**	−0.837 (0.038)*	−0.818 (0.046)*	−0.803 (0.054)
			M-CON_1_	0.601 (0.207)	0.640 (0.172)	−0.748 (0.087)	−0.735 (0.096)
			F-CON_1_	−0.178 (0.736)	0.027 (0.959)	−0.716 (0.110)	−0.262 (0.615)

*P < 0.05; **P < 0.01.

#### F1 generation

In the OF test, the peripheral time negatively correlated with FABP7 mRNA levels in 13M-LPS male mice (*r* = −0.873; *P* = 0.023) and negatively correlated with both FABP7 protein and mRNA levels in 13M-LPS female mice (*r* = −0.916, −0.920; *P* = 0.010, 0.009). However, the squares crossed positively correlated with FABP7 protein and mRNA levels in the 13M-LPS-M, 13M-LPS-F, and 13M-CON-M groups (all *P* < 0.05). In the BWA test, the latency before entering the white alley negatively correlated with levels of FABP7 protein and mRNA in the 13M-LPS male mice (*r* = −0.914, −0.888; *P* = 0.011, 0.018). Moreover, the BWA latency negatively correlated with FABP7 protein levels in 13M-CON female mice (*r* = −0.819, *P* = 0.046), and it negatively correlated with FABP7 mRNA levels in 13M-CON male mice (*r* = −0.976, *P* = 0.001). The time spent in the black alley negatively correlated with FABP7 protein and mRNA levels in three groups (LPS-M, CON-M, CON-F) at 13 months of age (all *P* < 0.05).

#### F2 generation

In the OF task, the peripheral time negatively correlated with FABP7 mRNA levels in the 3M F-LPS_1_ female mice (*r* = −0.834, *P* = 0.039) and with FABP7 protein levels in the 3M P-LPS_2_ male mice and 13M M-LPS_1_ female mice (*r* = −0.896, −0.945; *P* = 0.016, 0.004). Additionally, the peripheral time showed significant negative correlations with FABP7 protein and mRNA levels in the 3M M-LPS_1_ mice (all* P* < 0.05) and 13M M-CON_1_ female mice (*r* = −0.939, −0.843; *P* = 0.005, 0.035). The squares crossed positively correlated with both FABP7 protein and mRNA in two groups (M-LPS_1_, M-CON_1_) at 3 months of age (all *P* < 0.05) and in three groups (P-LPS_2_, M-LPS_1,_ F-LPS_1_) at 13 months of age (all *P* < 0.05), while it positively correlated with FABP7 protein in the 3M F-LPS_1_ female mice (*r* = 0.835; *P* = 0.039). In the BWA test, the latency to enter the white alley negatively correlated with FABP7 mRNA in the 3M F-LPS_1_ and 13M M-LPS_1_ female mice (*r* = −0.835, −0.913; *P* = 0.039, 0.011) and with protein levels in the 3M M-LPS_1_ and 13M-P-LPS_2_ male mice (*r* = −0.842, −0.978; *P* = 0.035, 0.001). The latency to enter the white alley showed significant negative correlations with FABP7 protein and mRNA levels in the 13M M-LPS_1_ male mice (all*P* < 0.05). A significant negative correlation occurred between the time spent in the black alley and FABP7 protein levels in the 3M F-CON_1_ female mice and 13M F-LPS_1_ male mice (*r* = −0.927, −0.818; *P* = 0.008, 0.046). Moreover, the time spent in the black alley negatively correlated with both FABP7 protein and mRNA in the 3M M-LPS_1_ male mice, 13M M-LPS_1_ mice, and 13M F-LPS_1_ female mice (all* P* < 0.05).

## Discussion

Prenatal inflammation has been associated with many neuropsychiatric disorders, such as anxiety (Depino, 2015; Hsueh et al., 2017). Programming by means of adverse “*in utero*” environmental factors can result in behavioral alterations that can be inherited over multiple generations *via* paternal and maternal transmission (Constantinof et al., 2016). We speculated that prenatal inflammation has intergenerational transmission effects on age-related anxiety-like behaviors in F2-generation mice. The present study aimed to examine how germline exposure to inflammation (i.e., within the developing F1 embryos) alters anxiety levels in adolescence and midlife in male and female F2 offspring as well as whether F1 mice exposed to prenatal inflammation mating with an unexposed mouse affects the anxiety-like behaviors of their offspring. The present study expanded upon previous findings to demonstrate that the maternal (F0 generation) gestational LPS exposure had the potential to alter not only the individual directly exposed *in utero* but also their offspring. This work explored wider intergenerational influences and further questions regarding lineage effects on age-related anxiety-like behaviors and FABP7 protein and mRNA expression.

### Effects of F0-generation gestational inflammation on anxiety-like behaviors in F1 and F2 generations

In the present study, middle-aged (13-month-old) CD-1 mice showed increased anxiety-like behaviors in the OF and BWA tasks compared to adolescent (3-month-old) mice but no differences in performance in the EPM task. The increase in age-related anxiety levels reflected in the finding was consistent with our previous study (Li et al., 2016), but the task-specific in this increase differed from the past, which is probably due to the anxiety-like behaviors evaluated in midlife.

Our results revealed that maternal (F0 generation) LPS exposure during late gestation could affect task- and sex-specific anxiety in F1 and F2 generations. In middle-aged F1 mice, maternal (F0 generation) gestational inflammation augmented the age-related increase in anxiety-like behaviors in the OF and EPM tasks. The OF task is usually used for the evaluation of exploratory, locomotor activity, and anxiety in rodents. Anxious animals tend to spend more time in the periphery, avoiding the center of the OF; peripheral time and latency were analyzed to estimate unconditioned anxiety-like behaviors in response to a novel environment. However, in the EPM task, the effect of LPS-affected mainly affected the time spent on the open arm. In F2 mice, the offspring from single or both LPS-affected parents showed more anxiety-like behaviors than the offspring from unaffected mothers or fathers in the OF and BWA tasks. Overall, the effect of prenatal inflammation on anxiety-like behaviors was maintained, especially in the offspring of maternal line. Furthermore, the F2 female offspring from LPS-affected mothers showed higher anxiety levels relative to the male offspring.

In F2 offspring, increased anxiety-like behaviors were observed at both 3 and 13 months of age, indicating that pathological anxiety that appears at a young age may persist into midlife. Compared to the female offspring from LPS-affected mothers and fathers, the F2 females from LPS-affected parents showed fewer squares crossed in the OF task in adolescence, while the males did not. Additionally, the F2 females from LPS-affected parents exhibited shorter OF peripheral time while longer BWA latencies in midlife. These results suggested that when both F1 parents were affected, the effects of prenatal inflammation on locomotor activity and anxiety were strengthened in sex- and task-specific manners relative to when only one parent was affected.

The adolescent F2 offspring manifested high anxiety levels and enhanced locomotor activity, though these were not observed in adolescent F1 offspring, indicating that even if parents exposed to *in utero* inflammation are asymptomatic for anxiety, they are able to transmit the anxiety phenotype to their offspring. One possibility may be that F1 animals do not show any obvious phenotype in adolescence because they were exposed to inflammation only in the embryonic period (GD15–17), unlike the F2 offspring, which carry the potential epigenetic changes starting in the germ cells across both pre- and postnatal development. Alternatively, since adaptive mechanisms in epigenetic inheritance or multiple physiological and molecular changes are likely induced in the embryonic stage, which can compensate for some deficits induced by prenatal inflammation in the F1 generation (Kirsten et al., 2012), this results in failure to transmit these phenotypes to the next generation (Franklin et al., 2011).

### Effects of gestational inflammation exposure in the F0 generation on hippocampal *Fabp7* expression in F1 and F2 generations

The hippocampus is the key brain region underlying anxiety in both clinical and experimental studies (Richardson et al., 2004; Engin and Treit, 2007; Keller et al., 2018). Astrocytes play important roles in the regulation of synaptic function, extracellular homeostasis, and hippocampal neurogenesis (Campos et al., 2020). The *Fabp7* gene, whose product FABP7 is highly expressed in hippocampal astrocytes and decreases significantly from early adulthood. FABP7 is involved in the proliferation of astrocytes (Sharifi et al., 2011), and plays a key role in neurogenesis *via* the maintenance of the pool of neural stem/progenitor cells (Watanabe et al., 2007). In the present study, a significant effect of age on hippocampal *Fabp7* expression was observed, the control middle-aged mice had lower FABP7 mRNA expression levels compared to corresponding adolescent mice.

For the intergenerational effects of prenatal inflammation on hippocampal *Fabp7* gene expression, our study revealed that maternal LPS exposure during late gestation could affect *Fabp7* expression levels in the F1 and F2 generations. For the F1 generation, the hippocampal FABP7 protein levels significantly decreased in both adolescence and midlife, whereas the mRNA levels were not affected at both ages. For the F2 generation, in adolescence, the effects of F0-generation LPS exposure were only observed in the offspring from the maternal lineage; namely, the offspring from LPS-affected mothers showed lower *Fabp7* expression than those from unaffected mothers. However, the intergenerational effects on *Fabp7* expression in the paternal lineage were not manifested until midlife. In the middle-aged F2 mice, the hippocampal *Fabp7* expression in both maternal and paternal lineages were influenced, though the effect was mainly attributed to the female mice. Furthermore, the intergenerational effect on middle-aged mice was sex-specific; namely, the female offspring from LPS-affected mothers or fathers showed significantly lower FABP7 protein and mRNA expression than the males from the same lineage. Unexpectedly, compared to the F2 offspring from single affected parents, *Fabp7* expression was not further decreased in the offspring from both parents were affected, suggesting that maternal and paternal conditions may independently affect *Fabp7* expression in the offspring. As such, the combined effects of maternal and paternal prenatal inflammation exposure did not produce greater effects than either alone.

### Changed expression of hippocampal *Fabp7* was associated with increased anxiety-like behaviors in F1 and F2 generations

In the current study, the effects of F0-generation LPS exposure on anxiety-like behaviors and *Fabp7* gene expression in F1 and F2 offspring were age-dependent and sex-specific. Importantly, increased anxiety-like behaviors might be related to altered levels of hippocampal *Fabp7* expression. In the OF task, a negative correlation was observed between peripheral time and hippocampal FABP7 protein levels and a positive correlation was observed between the squares crossed and FABP7 protein. In the BWA task, the latency before entering the white alley and the time spent in the black alley negatively correlated with the FABP7 protein levels (Tables s 3, 4). Moreover, the pattern of correlations between anxiety levels and FABP7 mRNA levels was similar. The current findings supported the idea that FABP7 levels showed a genetic association with anxiety levels, and the alteration of *Fabp7* gene expression may occur at the levels of transcription and/or translation. The intergenerational effect of increased anxiety-like behaviors in F2 offspring may be attributable to decreased *Fabp7* transcription, which then leads to decreased translation. For the F1 offspring, the timing of prenatal inflammation exposure and/or the nature of the inflammogen severally affect hippocampal neurogenesis (Meyer et al., 2006); offspring show reduced neurogenesis when inflammation occurs late in gestation (GD15–17; Zhang and van Praag, 2015). Furthermore, the late development of the hippocampus, especially of the dentate gyrus subregion, is more sensitive to late gestational immune activation than to earlier challenges (Depino, 2015). Neural stem cells and progenitor cells in the dentate gyrus subregion continuously generate new neurons after birth, and FABP7 is vital in the proliferation and survival of the neural stem and progenitor cells during postnatal hippocampal neurogenesis in mice (Matsumata et al., 2012). Therefore, F0 LPS exposure during late gestation may significantly influence hippocampal neurogenesis in F1 mice by reducing *Fabp7* expression, thereby increasing age-related anxiety-like behaviors.

The maternal immune activation (MIA) mouse model has been widely employed to demonstrate the effects of gestational infection on offspring emotionality. When a dam (F0 generation) was exposed to immunogens, both her children (F1, exposed as fetus) and grandchildren (F2, exposed as developing germ cells within the F1 fetus) were affected. In this case, exposing the fetus to an inflammatory environment also exposes developing germ cells, resulting in indirect “exposure” of its offspring as well. In Poly (I:C) MIA model in mice, intergenerational and transgenerational effects on emotional behaviors have been reported (Ronovsky et al., 2017; Berger et al., 2018), and the transgenerational effects on social and fear-related behaviors are mediated *via* the paternal lineage (Weber-Stadlbauer et al., 2017). In our study, increased anxiety-like behaviors in the F2 generation were observed in the LPS MIA model. Surprisingly, the intergenerational effects were mainly transferred in the maternal lineage and seemed to affect female offspring to a greater extent. We speculated that prenatal inflammation may increase the susceptibility of germ cells to anxiety *via* the influence of *Fabp7* expression. However, sex-dependent intergenerational transmission usually occurs in a complex manner. Maternal transmission in rodents provides indirect mechanistic insight into germ line contribution because females participate in the development and rearing processes rather than merely providing gametes (Rando, 2012). It remains unclear whether other possible factors exist in the effects of prenatal inflammation along the maternal line (Champagne, 2008; Yao et al., 2014). Even so, it is still critical to assess the phenotypic outcomes and transmission effects in both males and females and to undertake longitudinal analyses of outcomes to determine the long-term impacts of prenatal inflammation exposure.

### Summary

Collectively, the current study is the first to report that gestational (F0 generation) LPS exposure could augment age-related anxiety-like behavioral changes in both F1 and F2 generations, and these effects were possibly related to decreased *Fabp7* expression in the hippocampus, but we were not able to determine more conclusively the link between FABP7 and anxiety-like behaviors in the present study, further exploration is needed in the future. To truly establish the role of FABPs in both paternal and maternal transmission of prenatal inflammation effects, future studies must investigate the mechanisms by which these alterations are inherited from one generation to the next and the potential role of FABPs in phenotypic outcomes.

## Data Availability Statement

The raw data supporting the conclusions of this article will be made available by the authors, without undue reservation.

## Ethics Statement

The animal study was reviewed and approved by Association of Laboratory Animal Sciences and the Center for Laboratory Animal Sciences at Anhui Medical University.

## Author Contributions

JC and Z-ZZ conceived and designed the study and drafted the manuscript. B-LL and Q-GY carried out the immunohistochemistry and behavioral tests. M-ZN, Q-TW, and YL participated in the study design and statistical analysis. G-HC and X-WL guided the study, revised the manuscript, and take responsibility for the integrity of the data and the accuracy of the data analysis. All authors contributed to the article and approved the submitted version.

## Funding

This work was financially supported by the National Natural Science Foundation of China (81370444, 81671316), the Scientific Research Fund Project of Hunan Provincial Health Commission (20200497), and the Natural Science Foundation of Hunan Province of China (2021JJ70040).

## References

[B1] Adams R. C. M. Smith C. (2020). *In utero* exposure to maternal chronic inflammation transfers a pro-inflammatory profile to generation F2 via sex-specific mechanisms. Front. Immunol. 11:48. 10.3389/fimmu.2020.00048 32117231 PMC7031653

[B2] Adams R. C. M. Smith C. (2019). Chronic gestational inflammation: transfer of maternal adaptation over two generations of progeny. Mediators Inflamm. 2019:9160941. 10.1155/2019/9160941 31582905 PMC6754931

[B3] Babri S. Doosti M. H. Salari A. A. (2014). Strain-dependent effects of prenatal maternal immune activation on anxiety- and depression-like behaviors in offspring. Brain Behav. Immun. 37, 164–176. 10.1016/j.bbi.2013.12.003 24326014

[B4] Batinić B. Santrač A. Divović B. Timić T. Stanković T. Obradović A. L.. (2016). Lipopolysaccharide exposure during late embryogenesis results in diminished locomotor activity and amphetamine response in females and spatial cognition impairment in males in adult, but not adolescent rat offspring. Behav. Brain Res. 299, 72–80. 10.1016/j.bbr.2015.11.025 26620494

[B5] Benarroch E. E. (2013). Cation-chloride cotransporters in the nervous system: general features and clinical correlations. Neurology 80, 756–763. 10.1212/WNL.0b013e318283bb1c 23420893

[B6] Berger S. Ronovsky M. Horvath O. Berger A. Pollak D. D. (2018). Impact of maternal immune activation on maternal care behavior, offspring emotionality and intergenerational transmission in C3H/He mice. Brain Behav. Immun. 70, 131–140. 10.1016/j.bbi.2018.02.008 29481858

[B7] Bollati V. Baccarelli A. (2010). Environmental epigenetics. Heredity (Edinb). 105, 105–112. 10.1038/hdy.2010.2 20179736 PMC3133724

[B8] Boneva N. B. Kaplamadzhiev D. B. Sahara S. Kikuchi H. Pyko I. V. Kikuchi M.. (2011). Expression of fatty acid-binding proteins in adult hippocampal neurogenic niche of postischemic monkeys. Hippocampus 21, 162–171. 10.1002/hipo.20732 20014382

[B9] Brown A. S. Meyer U. (2018). Maternal immune activation and neuropsychiatric illness: a translational research perspective. Am. J. Psychiatry 175, 1073–1083. 10.1176/appi.ajp.2018.17121311 30220221 PMC6408273

[B10] Campos J. Guerra-Gomes S. Serra S. C. Baltazar G. Oliveira J. F. Teixeira F. G.. (2020). Astrocyte signaling impacts the effects of human bone marrow mesenchymal stem cells secretome application into the hippocampus: a proliferation and morphometrical analysis on astrocytic cell populations. Brain Res. 1732:146700. 10.1016/j.brainres.2020.146700 32032613

[B11] Champagne F. A. (2008). Epigenetic mechanisms and the transgenerational effects of maternal care. Front. Neuroendocrinol. 29, 386–397. 10.1016/j.yfrne.2008.03.003 18462782 PMC2682215

[B12] Chen G. H. Wang C. Yangcheng H. Y. Liu R. Y. Zhou J. N. (2007). Age-related changes in anxiety are task-specific in the senescence-accelerated prone mouse 8. Physiol. Behav. 91, 644–651. 10.1016/j.physbeh.2007.03.023 17481677

[B13] Chen G. H. Wang Y. J. Zhang L. Q. Zhou J. N. (2004). Age- and sex-related disturbance in a battery of sensorimotor and cognitive tasks in Kunming mice. Physiol. Behav. 83, 531–541. 10.1016/j.physbeh.2004.09.012 15581676

[B14] Chesnokova V. Pechnick R. N. Wawrowsky K. (2016). Chronic peripheral inflammation, hippocampal neurogenesis and behavior. Brain Behav. Immun. 58, 1–8. 10.1016/j.bbi.2016.01.017 26802985 PMC4956598

[B15] Coley E. J. L. Demaestri C. Ganguly P. Honeycutt J. A. Peterzell S. Rose N.. (2019). Cross-generational transmission of early life stress effects on HPA regulators and Bdnf are mediated by sex, lineage and upbringing. Front. Behav. Neurosci. 13:101. 10.3389/fnbeh.2019.00101 31143105 PMC6521572

[B16] Constantinof A. Moisiadis V. G. Matthews S. G. (2016). Programming of stress pathways: a transgenerational perspective. J. Steroid Biochem. Mol. Biol. 160, 175–180. 10.1016/j.jsbmb.2015.10.008 26474822

[B17] Depino A. M. (2015). Early prenatal exposure to LPS results in anxiety- and depression-related behaviors in adulthood. Neuroscience 299, 56–65. 10.1016/j.neuroscience.2015.04.065 25943476

[B18] Depino A. Ferrari C. Pott Godoy M. C. Tarelli R. Pitossi F. J. (2005). Differential effects of interleukin-1beta on neurotoxicity, cytokine induction and glial reaction in specific brain regions. J. Neuroimmunol. 168, 96–110. 10.1016/j.jneuroim.2005.07.009 16112750

[B19] Drevets W. C. Price J. L. Furey M. L. (2008). Brain structural and functional abnormalities in mood disorders: implications for neurocircuitry models of depression. Brain Struct. Funct. 213, 93–118. 10.1007/s00429-008-0189-x 18704495 PMC2522333

[B20] Enayati M. Solati J. Hosseini M. H. Shahi H. R. Saki G. Salari A. A. (2012). Maternal infection during late pregnancy increases anxiety- and depression-like behaviors with increasing age in male offspring. Brain Res. Bull. 87, 295–302. 10.1016/j.brainresbull.2011.08.015 21893170

[B21] Encinas J. M. Michurina T. V. Peunova N. Park J. H. Tordo J. Peterson D. A.. (2011). Division-coupled astrocytic differentiation and age-related depletion of neural stem cells in the adult hippocampus. Cell Stem Cell 8, 566–579. 10.1016/j.stem.2011.03.010 21549330 PMC3286186

[B22] Engin E. Treit D. (2007). The role of hippocampus in anxiety: intracerebral infusion studies. Behav. Pharmacol. 18, 365–374. 10.1097/FBP.0b013e3282de7929 17762507

[B23] Estes M. L. McAllister A. K. (2016). Maternal immune activation: implications for neuropsychiatric disorders. Science 353, 772–777. 10.1126/science.aag3194 27540164 PMC5650490

[B24] Foerster S. Guzman de la Fuente A. Kagawa Y. Bartels T. Owada Y. Franklin R. J. M. (2020). The fatty acid binding protein FABP7 is required for optimal oligodendrocyte differentiation during myelination but not during remyelination. Glia 68, 1410–1420. 10.1002/glia.23789 32017258 PMC7317849

[B25] Franklin T. B. Linder N. Russig H. Thöny B. Mansuy I. M. (2011). Influence of early stress on social abilities and serotonergic functions across generations in mice. PLoS One 6:e21842. 10.1371/journal.pone.0021842 21799751 PMC3143115

[B26] Furuhashi M. Hotamisligil G. S. (2008). Fatty acid-binding proteins: role in metabolic diseases and potential as drug targets. Nat. Rev. Drug Discov. 7, 489–503. 10.1038/nrd2589 18511927 PMC2821027

[B27] Fuss J. Ben Abdallah N. M. Vogt M. A. Touma C. Pacifici P. G. Palme R.. (2010). Voluntary exercise induces anxiety-like behavior in adult C57BL/6J mice correlating with hippocampal neurogenesis. Hippocampus 20, 364–376. 10.1002/hipo.20634 19452518

[B28] Gerstner J. R. Bremer Q. Z. Vander Heyden W. M. Lavaute T. M. Yin J. C. Landry C. F. (2008). Brain fatty acid binding protein (Fabp7) is diurnally regulated in astrocytes and hippocampal granule cell precursors in adult rodent brain. PLoS One 3:e1631. 10.1371/journal.pone.0001631 18286188 PMC2238817

[B29] Giachino C. Basak O. Lugert S. Knuckles P. Obernier K. Fiorelli R.. (2014). Molecular diversity subdivides the adult forebrain neural stem cell population. Stem Cells 32, 70–84. 10.1002/stem.1520 23964022 PMC4259462

[B30] Green H. F. Nolan Y. M. (2014). Inflammation and the developing brain: consequences for hippocampal neurogenesis and behavior. Neurosci. Biobehav. Rev. 40, 20–34. 10.1016/j.neubiorev.2014.01.004 24462889

[B31] Hanamsagar R. Bilbo S. D. (2016). Sex differences in neurodevelopmental and neurodegenerative disorders: focus on microglial function and neuroinflammation during development. J. Steroid Biochem. Mol. Biol. 160, 127–133. 10.1016/j.jsbmb.2015.09.039 26435451 PMC4829467

[B32] Hsueh P. T. Wang H. H. Liu C. L. Ni W. F. Chen Y. L. Liu J. K. (2017). Expression of cerebral serotonin related to anxiety-like behaviors in C57BL/6 offspring induced by repeated subcutaneous prenatal exposure to low-dose lipopolysaccharide. PLoS One 12:e0179970. 10.1371/journal.pone.0179970 28650979 PMC5484498

[B33] Insel T. R. Wang P. S. (2010). Rethinking mental illness. JAMA 303, 1970–1971. 10.1001/jama.2010.555 20483974

[B34] Keller D. Erö C. Markram H. (2018). Cell densities in the mouse brain: a systematic review. Front. Neuroanat. 12:83. 10.3389/fnana.2018.00083 30405363 PMC6205984

[B35] Kinsella M. T. Monk C. (2009). Impact of maternal stress, depression and anxiety on fetal neurobehavioral development. Clin. Obstet. Gynecol. 52, 425–440. 10.1097/GRF.0b013e3181b52df1 19661759 PMC3710585

[B36] Kirsten T. B. Chaves-Kirsten G. P. Chaible L. M. Silva A. C. Martins D. O. Britto L. R.. (2012). Hypoactivity of the central dopaminergic system and autistic-like behavior induced by a single early prenatal exposure to lipopolysaccharide. J. Neurosci. Res. 90, 1903–1912. 10.1002/jnr.23089 22714803

[B37] Koga M. Nakagawa S. Sato A. Oka M. Makikhara K. Sakai Y.. (2021). Plasma fatty acid-binding protein 7 concentration correlates with depression/anxiety, cognition and positive symptom in patients with schizophrenia. J. Psychiatr. Res. 144, 304–311. 10.1016/j.jpsychires.2021.10.028 34715597

[B38] Li W. Chen M. Feng X. Song M. Shao M. Yang Y.. (2021). Maternal immune activation alters adult behavior, intestinal integrity, gut microbiota and the gut inflammation. Brain Behav. 11:e02133. 10.1002/brb3.2133 33793085 PMC8119836

[B39] Li X. Y. Wang F. Chen G. H. Li X. W. Yang Q. G. Cao L.. (2016). Inflammatory insult during pregnancy accelerates age-related behavioral and neurobiochemical changes in CD-1 mice. Age (Dordr) 38:59. 10.1007/s11357-016-9920-3 27194408 PMC5005951

[B40] Lin Y. L. Lin S. Y. Wang S. (2012). Prenatal lipopolysaccharide exposure increases anxiety-like behaviors and enhances stress-induced corticosterone responses in adult rats. Brain Behav. Immun. 26, 459–468. 10.1016/j.bbi.2011.12.003 22198119

[B41] Liu R. Z. Mita R. Beaulieu M. Gao Z. Godbout R. (2010). Fatty acid binding proteins in brain development and disease. Int. J. Dev. Biol. 54, 1229–1239. 10.1387/ijdb.092976rl 20563994

[B42] Mac Giollabhui N. Breen E. C. Murphy S. K. Maxwell S. D. Cohn B. A. Krigbaum N. Y.. (2019). Maternal inflammation during pregnancy and offspring psychiatric symptoms in childhood: timing and sex matter. J. Psychiatr. Res. 111, 96–103. 10.1016/j.jpsychires.2019.01.009 30690329 PMC6644717

[B43] Maekawa M. Owada Y. Yoshikawa T. (2011). Role of polyunsaturated fatty acids and fatty acid binding protein in the pathogenesis of schizophrenia. Curr. Pharm. Des. 17, 168–175. 10.2174/138161211795049615 21355837

[B44] Matsumata M. Inada H. Osumi N. (2016). Fatty acid binding proteins and the nervous system: their impact on mental conditions. Neurosci. Res. 102, 47–55. 10.1016/j.neures.2014.08.012 25205626

[B45] Matsumata M. Sakayori N. Maekawa M. Owada Y. Yoshikawa T. Osumi N. (2012). The effects of Fabp7 and Fabp5 on postnatal hippocampal neurogenesis in the mouse. Stem Cells 30, 1532–1543. 10.1002/stem.1124 22581784

[B46] Meyer U. Nyffeler M. Engler A. Urwyler A. Schedlowski M. Knuesel I.. (2006). The time of prenatal immune challenge determines the specificity of inflammation-mediated brain and behavioral pathology. J. Neurosci. 26, 4752–4762. 10.1523/JNEUROSCI.0099-06.2006 16672647 PMC6674174

[B47] Moorthi P. Premkumar P. Priyanka R. Jayachandran K. S. Anusuyadevi M. (2015). Pathological changes in hippocampal neuronal circuits underlie age-associated neurodegeneration and memory loss: positive clue toward SAD. Neuroscience 301, 90–105. 10.1016/j.neuroscience.2015.05.062 26045180

[B48] Mouihate A. Kalakh S. AlMutairi R. AlashQ18r A. (2019). Prenatal inflammation dampens neurogenesis and enhances serotonin transporter expression in the hippocampus of adult female rats. Med. Princ. Pract. 28, 352–360. 10.1159/000499658 30884483 PMC6639577

[B49] Murray K. N. Edye M. E. Manca M. Vernon A. C. Oladipo J. M. Fasolino V.. (2019). Evolution of a maternal immune activation (mIA) model in rats: early developmental effects. Brain Behav. Immun. 75, 48–59. 10.1016/j.bbi.2018.09.005 30218784

[B50] National Research Council (US) Committee for the Update of the Guide for the Care and Use of Laboratory Animals (2011). Guide for the Care and Use of Laboratory Animals, 8th ed. Washington, DC: National Academies Press (US). 21595115

[B51] O’Donnell K. J. Meaney M. J. (2017). Fetal origins of mental health: the developmental origins of health and disease hypothesis. Am. J. Psychiatry 174, 319–328. 10.1176/appi.ajp.2016.16020138 27838934

[B52] Penteado S. H. W. Teodorov E. Kirsten T. B. Eluf B. P. Reis-Silva T. M. Acenjo M. K.. (2014). Prenatal lipopolysaccharide disrupts maternal behavior, reduces nest odor preference in pups and induces anxiety: studies of F1 and F2 generations. Eur. J. Pharmacol. 738, 342–351. 10.1016/j.ejphar.2014.05.058 24927995

[B53] Rando O. J. (2012). Daddy issues: paternal effects on phenotype. Cell 151, 702–708. 10.1016/j.cell.2012.10.020 23141533 PMC3564497

[B54] Revest J. M. Dupret D. Koehl M. Funk-Reiter C. Grosjean N. Piazza P. V.. (2009). Adult hippocampal neurogenesis is involved in anxiety-related behaviors. Mol. Psychiatry 14, 959–967. 10.1038/mp.2009.15 19255582

[B55] Richardson M. P. Strange B. A. Dolan R. J. (2004). Encoding of emotional memories depends on amygdala and hippocampus and their interactions. Nat. Neurosci. 7, 278–285. 10.1038/nn1190 14758364

[B56] Ronovsky M. Berger S. Zambon A. Reisinger S. N. Horvath O. Pollak A.. (2017). Maternal immune activation transgenerationally modulates maternal care and offspring depression-like behavior. Brain Behav. Immun. 63, 127–136. 10.1016/j.bbi.2016.10.016 27765645

[B57] Rytova V. Ganella D. E. Hawkes D. Bathgate R. A. D. Ma S. Gundlach A. L. (2019). Chronic activation of the relaxin-3 receptor on GABA neurons in rat ventral hippocampus promotes anxiety and social avoidance. Hippocampus 29, 905–920. 10.1002/hipo.23089 30891856

[B58] Schneider C. A. Rasband W. S. Eliceiri K. W. (2012). NIH image to ImageJ: 25 years of image analysis. Nat. Methods 9, 671–675. 10.1038/nmeth.2089 22930834 PMC5554542

[B59] Schulz D. Huston J. P. Buddenberg T. Topic B. (2007). “Despair” induced by extinction trials in the water maze: relationship with measures of anxiety in aged and adult rats. Neurobiol. Learn. Mem. 87, 309–323. 10.1016/j.nlm.2006.09.006 17079170

[B60] Sharifi K. Morihiro Y. Maekawa M. Yasumoto Y. Hoshi H. Adachi Y.. (2011). FABP7 expression in normal and stab-injured brain cortex and its role in astrocyte proliferation. Histochem. Cell Biol. 136, 501–513. 10.1007/s00418-011-0865-4 21938553 PMC3192944

[B61] Shimamoto C. Ohnishi T. Maekawa M. Watanabe A. Ohba H. Arai R.. (2014). Functional characterization of FABP3, 5 and 7 gene variants identified in schizophrenia and autism spectrum disorder and mouse behavioral studies. Hum. Mol. Genet. 23, 6495–6511. 10.1093/hmg/ddu369 25027319 PMC4240203

[B62] Tsai S. F. Ku N. W. Wang T. F. Yang Y. H. Shih Y. H. Wu S. Y.. (2018). Long-term moderate exercise rescues age-related decline in hippocampal neuronal complexity and memory. Gerontology 64, 551–561. 10.1159/000488589 29734165

[B63] Wang F. Zhang Z. Z. Cao L. Yang Q. G. Lu Q. F. Chen G. H. (2020). Lipopolysaccharide exposure during late embryogenesis triggers and drives Alzheimer-like behavioral and neuropathological changes in CD-1 mice. Brain Behav. 10:e01546. 10.1002/brb3.1546 31997558 PMC7066339

[B64] Watanabe A. Toyota T. Owada Y. Hayashi T. Iwayama Y. Matsumata M.. (2007). Fabp7 maps to a quantitative trait locus for a schizophrenia endophenotype. PLoS Biol. 5:e297. 10.1371/journal.pbio.0050297 18001149 PMC2071943

[B65] Weber-Stadlbauer U. Richetto J. Labouesse M. A. Bohacek J. Mansuy I. M. Meyer U. (2017). Transgenerational transmission and modification of pathological traits induced by prenatal immune activation. Mol. Psychiatry 22, 102–112. 10.1038/mp.2016.41 27021823

[B66] Weissman M. M. Wickramaratne P. Adams P. Wolk S. Verdeli H. Olfson M. (2000). Brief screening for family psychiatric history: the family history screen. Arch. Gen. Psychiatry 57, 675–682. 10.1001/archpsyc.57.7.675 10891038

[B67] Yamamoto Y. Owada Y. (2021). Possible involvement of fatty acid binding proteins in psychiatric disorders. Anat. Sci. Int. 96, 333–342. 10.1007/s12565-020-00598-0 33604770

[B68] Yao Y. Robinson A. M. Zucchi F. C. Robbins J. C. Babenko O. Kovalchuk O.. (2014). Ancestral exposure to stress epigenetically programs preterm birth risk and adverse maternal and newborn outcomes. BMC Med. 12:121. 10.1186/s12916-014-0121-6 25286408 PMC4244860

[B69] Zhang Z. van Praag H. v. (2015). Maternal immune activation differentially impacts mature and adult-born hippocampal neurons in male mice. Brain Behav. Immun. 45, 60–70. 10.1016/j.bbi.2014.10.010 25449671

