# Supplementary material for: Prenatal exposure to inflammation increases anxiety-like behaviors in F1 and F2 generations: possible links to decreased FABP7 in hippocampus
Source: Front Behav Neurosci. 2022 Oct 10;16:973069. doi: 10.3389/fnbeh.2022.973069 (PMC9588974; doi:10.3389/fnbeh.2022.973069)
Supplement: Supplementary file 1 [file Table_1.DOCX]

**Supplementary Table S1**. Effects in F1 offspring in the battery of anxiety tasks

| Tasks | Measures | Age effect | | | | | | | |
| --- | --- | --- | --- | --- | --- | --- | --- | --- | --- |
|  |  | All mice | | CON-M | | | CON-F | | |
| EPM | NOA | **−** | | | **−** | | | **−** | |
|  | TOA | **−** | | | **−** | | | **−** | |
| OF | PT | **−** | | | **−** | | | **−** | |
|  | Latency | **↑** | | | **−** | | | **↑** | |
|  | NOL | **−** | | | **−** | | | **−** | |
| BWA | Latency | **↑** | | | **−** | | | **−** | |
|  | BT | **↑** | | | **↑** | | | **↑** | |
|  | NBW | **−** | | | **−** | | | **−** | |
| Tasks | Measures | LPS-affected effect of F1 | | | | | | | |
|  |  | 3 months | | | | 13 months | | | |
|  |  | All mice | 3mo-male | 3mo-female | | All mice | 13mo-male | | 13mo-female |
| EPM | NOA | **−** | **−** | **−** | | **−** | **−** | | **−** |
|  | TOA | **−** | **−** | **−** | | **↑** | **−** | | **↑** |
| OF | PT | **−** | **−** | **−** | | **↑** | **↑** | | **↑** |
|  | OF Latency | **−** | **−** | **−** | | **−** | **−** | | **−** |
|  | NOL | **−** | **−** | **−** | | **↓** | **↓** | | **↓** |
| BWA | Latency | **−** | **−** | **−** | | **−** | **−** | | **−** |
|  | BT | **−** | **−** | **−** | | **−** | **−** | | **−** |
|  | NBW | **−** | **−** | **−** | | **−** | **−** | | **−** |
| Tasks | Measures | sex effect of F1 | | | | | | | |
|  |  | 3 months | | | | 13 months | | | |
|  |  | All mice | 3mo-male | 3mo-female | | All mice | 13mo-male | | 13mo-female |
| EPM | NOA | **−** | **−** | **−** | | **−** | **−** | | **−** |
|  | TOA | **−** | **−** | **−** | | **−** | **−** | | **−** |
| OF | PT | **−** | **−** | **−** | | **−** | **−** | | **−** |
|  | OF Latency | **−** | **−** | **−** | | **−** | **−** | | **−** |
|  | NOL | **−** | **−** | **−** | | **↑** | **−** | | **−** |
| BWA | Latency | **−** | **−** | **−** | | **−** | **−** | | **−** |
|  | BT | **−** | **−** | **−** | | **↑** | **−** | | **↑** |
|  | NBW | **−** | **−** | **−** | | **↑** | **↑** | | **−** |

**Supplementary table 2.** Effects in F2 offspring in the battery of anxiety tasks

| Tasks | Measures | LPS-affected effect | | | | | | | | | | | | | | | | | | |
| --- | --- | --- | --- | --- | --- | --- | --- | --- | --- | --- | --- | --- | --- | --- | --- | --- | --- | --- | --- | --- |
|  |  | 3mo-male | | | | | | | | | | 3mo-female | | | | | | | | |
|  |  | All mice | | | P-LPS_2_ | | M-LPS_1_ | | | F-LPS_1_ | | P-LPS_2_ | | | M-LPS_1_ | | | F-LPS_1_ | | |
| EPM | NOA | **−** | | | **−** | | **−** | | | **−** | | **−** | | | **−** | | | **−** | | |
|  | TOA | **−** | | | **−** | | **−** | | | **−** | | **−** | | | **−** | | | **−** | | |
| OF | PT | **↑** | | | **−** | | **−** | | | **−** | | **−** | | | **↑** | | | **−** | | |
|  | OF Latency | **−** | | | **−** | | **−** | | | **−** | | **−** | | | **−** | | | **−** | | |
|  | NOL | **↓** | | | **−** | | **↓** | | | **−** | | **↓** | | | **−** | | | **−** | | |
| BWA | Latency | **−** | | | **−** | | **−** | | | **−** | | **−** | | | **−** | | | **−** | | |
|  | BT | **−** | | | **−** | | **−** | | | **−** | | **−** | | | **−** | | | **−** | | |
|  | NBW | **−** | | | **−** | | **−** | | | **−** | | **−** | | | **−** | | | **−** | | |
| Tasks | Measures | 13mo-male | | | | | | | | | | 13mo-female | | | | | | | | |
|  |  | All mice | | | P-LPS_2_ | | M-LPS_1_ | | | F-LPS_1_ | | P-LPS_2_ | | | M-LPS_1_ | | | F-LPS_1_ | | |
| EPM | NOA | **−** | | | **−** | | **−** | | | **−** | | **−** | | | **−** | | | **−** | | |
|  | TOA | **−** | | | **−** | | **−** | | | **−** | | **−** | | | **−** | | | **−** | | |
| OF | PT | **↑** | | | **−** | | **−** | | | **−** | | **↓** | | | **↑** | | | **↑** | | |
|  | Latency | **↑** | | | **−** | | **↑** | | | **−** | | **−** | | | **↑** | | | **−** | | |
|  | NOL | **↓** | | | **−** | | **−** | | | **−** | | **−** | | | **↓** | | | **−** | | |
| BWA | Latency | **↑** | | | **−** | | **−** | | | **−** | | **↑** | | | **−** | | | **−** | | |
|  | BT | **↑** | | | **−** | | **−** | | | **−** | | **−** | | | **↑** | | | **−** | | |
|  | NBW | **−** | | | **−** | | **−** | | | **−** | | **−** | | | **−** | | | **−** | | |
| Tasks | Measures | Sex effect | | | | | | | | | | | | | | | | | | |
|  |  | 3 months | | | | | | | | | 13 months | | | | | | | | | |
|  |  | All mice | P-LPS_2_ | M-LPS_1_ | | F-LPS_1_ | | M-CON_1_ | F-CON_1_ | | All mice | | P-LPS_2_ | M-LPS_1_ | | F-LPS_1_ | M-CON_1_ | | F-CON_1_ |  |
| EPM | NOA | **−** | **−** | **−** | | **−** | | **−** | **−** | | **−** | | **−** | **−** | | **−** | **−** | | **−** |  |
|  | TOA | **−** | **−** | **−** | | **−** | | **−** | **−** | | **−** | | **−** | **−** | | **−** | **−** | | **−** |  |
| OF | PT | **↑** | **−** | **↑** | | **−** | | **−** | **−** | | **−** | | **−** | **−** | | **−** | **−** | | **−** |  |
|  | OF Latency | **−** | **−** | **−** | | **−** | | **−** | **−** | | **−** | | **−** | **−** | | **−** | **−** | | **−** |  |
|  | NOL | **−** | **−** | **−** | | **−** | | **−** | **−** | | **−** | | **−** | **−** | | **−** | **−** | | **−** |  |
| BWA | Latency | **−** | **−** | **−** | | **−** | | **−** | **−** | | **−** | | **−** | **−** | | **−** | **−** | | **−** |  |
|  | BT | **−** | **−** | **−** | | **−** | | **−** | **−** | | **↑** | | **−** | **↑** | | **−** | **−** | | **−** |  |
|  | NBW | **−** | **−** | **−** | | **−** | | **−** | **−** | | **−** | | **−** | **−** | | **−** | **−** | | **−** |  |
